# Suppressor of variegation 3–9 homologue 1 impairment and neutrophil-skewed systemic inflammation are associated with comorbidities in COPD

**DOI:** 10.1186/s12890-021-01628-x

**Published:** 2021-10-02

**Authors:** Tzu-Tao Chen, Sheng-Ming Wu, Kuan-Yuan Chen, Chien-Hua Tseng, Shu-Chuan Ho, Hsiao-Chi Chuang, Po-Hao Feng, Wen-Te Liu, Chia-Li Han, Erick Wan-Chun Huang, Yun-Kai Yeh, Kang-Yun Lee

**Affiliations:** 1 Graduate Institute of Clinical Medicine, College of Medicine, Taipei Medical University, Taipei, Taiwan; 2 Division of Pulmonary Medicine, Department of Internal Medicine, Shuang Ho Hospital, Taipei Medical University, New Taipei City, Taiwan; 3 Division of Pulmonary Medicine, Department of Internal Medicine, School of Medicine, College of Medicine, Taipei Medical University, Taipei, Taiwan; 4 School of Respiratory Therapy, College of Medicine, Taipei Medical University, Taipei, Taiwan; 5 Master Program in Clinical Pharmacogenomics and Pharmacoproteomics, College of Pharmacy, Taipei, Taiwan; 6 Woolcock Institute of Medical Research, Sydney, Australia; 7 South Western Sydney Clinical School, University of New South Wales, Sydney, Australia

**Keywords:** COPD, Neutrophil, Comorbidity, Inflammation, SUV39H1

## Abstract

**Background:**

Systemic manifestations and comorbidities are characteristics of chronic obstructive pulmonary disease (COPD) and are probably due to systemic inflammation. The histone methyltransferase SUV39H1 controls the Th1/Th2 balance. We previously reported that reduced SUV39H1 expression contributed to abnormal inflammation in COPD. Here, we aimed to determine whether impaired SUV39H1 expression in COPD patients associated with neutrophilic/eosinophilic inflammation responses and comorbidities.

**Methods:**

A total of 213 COPD patients and 13 healthy controls were recruited from the Shuang Ho Hospital, Taipei Medical University. SUV39H1 levels in peripheral blood mononuclear cells (PBMCs) from 13 healthy and 30 COPD participants were measured by immunoblotting. We classified the patients into two groups based on low (fold change, FC < 0.5) and high SUV39H1 expression (FC ≥ 0.5) compared to normal controls. Clinical outcomes including neutrophil or eosinophil counts associated with SUV39H1-related inflammation were evaluated by Chi square analyses or Mann–Whitney U test. The correlations between the percentage of neutrophils and number of COPD comorbidities or Charlson Comorbidity Index (CCI) scores were performed by Spearman’s rank analysis.

**Results:**

Low SUV39H1 expression group had high neutrophil counts relative to high SUV39H1expression group. In the COPD cohort, the high comorbidity group (≥ 2 comorbidities) had higher counts of whole white blood cell (WBC) and neutrophil, and lower proportion of eosinophil and eosinophil/neutrophil, as compared with low comorbidity group (0 and 1 comorbidities). The quantity of neutrophils was associated with COPD comorbidities (Spearman's *r* = 0.388, *p* < 0.001), but not with CCI scores. We also found that the high comorbidity group had more exacerbations per year compared with low comorbidity group (1.5 vs. 0.9 average exacerbations, *p* = 0.005). However, there were no significant differences between groups with these non-frequent (0–1 exacerbation) and frequent exacerbations per year (> 1 exacerbation) in numbers of WBC and proportion of neutrophils, eosinophils or eosinophil/neutrophil. Finally, patients with high comorbidities had lower SUV39H1 levels in their PBMCs than did those with low comorbidities.

**Conclusion:**

Blood neutrophil counts are associated with comorbidities in COPD patients. Impaired SUV39H1 expression in PBMCs from COPD patients are correlated with neutrophilic inflammation and comorbidities.

**Supplementary Information:**

The online version contains supplementary material available at 10.1186/s12890-021-01628-x.

## Background

Chronic obstructive pulmonary disease (COPD) is a chronic inflammatory airway disease with systemic manifestations and comorbidities, e.g., osteoporosis, hyperglycaemia, cardiovascular dysfunction, and even different malignant neoplasms [1]. The comorbidities are believed to be related to systemic inflammation. The accompanying progression of comorbidities has a large contribution to prolonged hospitalization, leading to increased medical expenses and overall mortality [2]. Although current pharmacological therapy, which is mainly focused treating on airway inflammation and airflow limitation, effectively relieves symptoms, improves the health status, and reduces exacerbations, a decrease in mortality has not been confirmed. Two large-scale clinical trials using inhaled corticosteroids plus long-acting beta2-agonists failed their primary endpoint of mortality [3, 4]. In addition to developing more effective treatments for airway issue, a strategy to combat systemic effects, probably by targeting systemic inflammation, is urgently needed.

Although nonspecific inflammation and the Th1 response have been generally recognized as the major sources of inflammation in COPD, Th2 effector eosinophils are also implicated in some patients. Eosinophilic COPD, which is defined using peripheral blood eosinophils as a practical biomarker, is a generally accepted treatable trait that is responsive to inhaled corticosteroids [5]. Recent meta-analyses [6] have clearly demonstrated a continuous relationship between ICS prescription and a reduced exacerbation risk at variable thresholds, confirming the biomarker role of blood eosinophils. Nevertheless, anti-IL5 or anti-IL-5 receptor monoclonal antibodies have failed to demonstrate consistent effects on patients with high blood eosinophil counts [7]. Blood eosinophils are also associated with a trend towards an increased risk of exacerbation, with either a positive relationship or no relationship [5]. Interestingly, in the Hokkaido COPD Cohort Study, subjects with blood eosinophilia had significantly slower annual FEV1 decline rates [8]. Additionally, subjects with two or more asthma-like features had a lower 10-year mortality rate. Therefore, the role of eosinophils in COPD is more complicated than that in asthma. Understanding the control of eosinophilia in COPD is therefore critical.

The histone methyltransferase SUV39H1 mediates the epigenetic silencing pathway controlling the Th1 response and maintains the balance between Th1 and Th2 responses [9]. Previously, we reported that reduced SUV39H1 expression was implicated in the abnormal inflammation of COPD observed in the clinical setting [10]. The reduction in SUV39H1 expression was correlated with poor pulmonary function and elevated serum levels of IL-6 and IL-8. We thus hypothesized that impaired SUV39H1 expression in COPD patients leads to neutrophilic and Th1-skewed inflammation and therefore reduced eosinophilia. We also studied the relationships of SUV39H1 and related inflammation with major clinical outcomes to understand their clinical impacts.

## Methods

### Study population

A cohort study was conducted in a tertiary teaching hospital in New Taipei City. Patients with COPD were diagnosed and graded according to the guidelines of the Global Initiative for Obstructive Lung Disease [11]. Normal subjects and patients with COPD, who were aged between 40 and 80 years old and signed an informed consent form, were enrolled during the study period from 2015 to 2017. Total blood and peripheral blood mononuclear cells (PBMCs) were harvested from the healthy subjects and patients with stable COPD. For a retrospective observational study (213 COPD patients), we reviewed the medical records of patients in our COPD registry, which were obtained from both outpatient clinics and inpatient wards, from March 2015 to December 2017. The study protocol was approved by the Taipei Medical University-Joint Institutional Review Board (TMU-JIRB N201502024 and N201802023). All experiments were performed in accordance with the relevant guidelines and regulations.

The COPD registry enrolled patients with a diagnosis of COPD confirmed at least twice within 90 days and a pulmonary function test compatible with a post-bronchodilator FEV1/FVC ratio < 70%. The timing of the haemogram obtained was defined as follows: 1. No recent bacterial infection within 7 days, 2. No systemic steroid use within 1 month, 3. No previous exacerbation within 3 months, and 4. No chemotherapy administration within 2 weeks. Total WBC and automated differential counts were measured with a haematology analyser (UniCel DxH 800 cellular analysis system, Beckman Coulter, Miami, FL, USA).

An exacerbation of COPD was counted only if it was a moderate or severe exacerbation, which is defined by the 2019 report of the Global Initiative for Chronic Obstructive Lung Disease as patients being treated with a short-acting bronchodilator plus antibiotics or oral corticosteroids or patients requiring hospitalization, visiting the emergency room or exhibiting acute respiratory failure. The comorbidities that were strongly related to COPD were defined according to the comprehensive review article by Professor Peter John Barnes published in 2009 [1] and included heart failure, coronary artery disease, pulmonary hypertension, lung cancer, anxiety/depression, osteoporosis, malnutrition, diabetes mellitus, obstructive sleep apnoea, normocytic anaemia, and lung fibrosis. We also compared our results with these specific comorbities and Charlson comorbidity index (CCI) score, which was first described by Mary Charlson in 1987 for prediction of 1 year mortality in specific comorbidities [12]. The calculation of CCI is the sum of all of the following items: age 50–59 years (1 point), 60–69 years (2 points), 70–79 years (3 points), age ≥ 80 years (4 points); myocardial infarction (1 point); congestive heart failure (1 point); peripheral vascular disease (1 point); history of cerebrovascular accident (1 point); dementia (1 point); COPD (1 point); connective tissue disease (1 point); peptic ulcer disease (1 point); liver disease (chronic hepatitis or cirrhosis without portal hypertension (1 point), cirrhosis with portal hypertension (3 points)); diabetes mellitus [uncomplicated (1 point), with end-organ damagen (2 points)]; hemiplegia (2 points); moderate to severe chronic kidney disease (2 points); solid organ tumor (localized (2 points), metastatic (6 points)); leukemia (2 points); lymphoma (2 points); AIDS (6 points).

### Western blot analysis

PBMCs from normal or COPD subjects were separated from the whole blood by Ficoll-Hypaque density gradient centrifugation, as previously described [13]. Total cellular proteins (30 μg) were subjected to 10% SDS-polyacrylamide gel electrophoresis and blotted onto polyvinylidene difluoride membranes. Antibodies against SUV39H1 (Cell Signalling, Hitchin, UK) and β-actin (Abcam, Cambridge, UK) were used for immunoblotting. The levels of immunoreactive bands were measured using Image Gauge software (Fuji Film, Tokyo, Japan). The fold change (FC) < 0.5 or FC ≥ 0.5 of SUV39H1 proteins in COPD patients were indicated as groups of low (SUV-Lo) or high (SUV-Hi) SUV39-H1 expression relative to average normal controls, respectively.

### Statistical analysis

Data were analysed with GraphPad Prism 5.0 software (GraphPad Software, San Diego, CA, USA). Unless stated otherwise, all data except age and BMI (which were naturally distributed) are presented as median and interquartile range (IQR). Chi square analyses were compared for Cigarette smoking, Comorbidities and COPD subtypes and Mann–Whitney U test for other variables. Spearman’s rank correlation was used to compare the relations between the percentage of neutrophils and number of COPD comorbidities or CCI. Differences were considered significant at *p* < 0.05.

## Results

### Low SUV39H1 expression was associated with high blood neutrophil counts

**Fig. 1:**
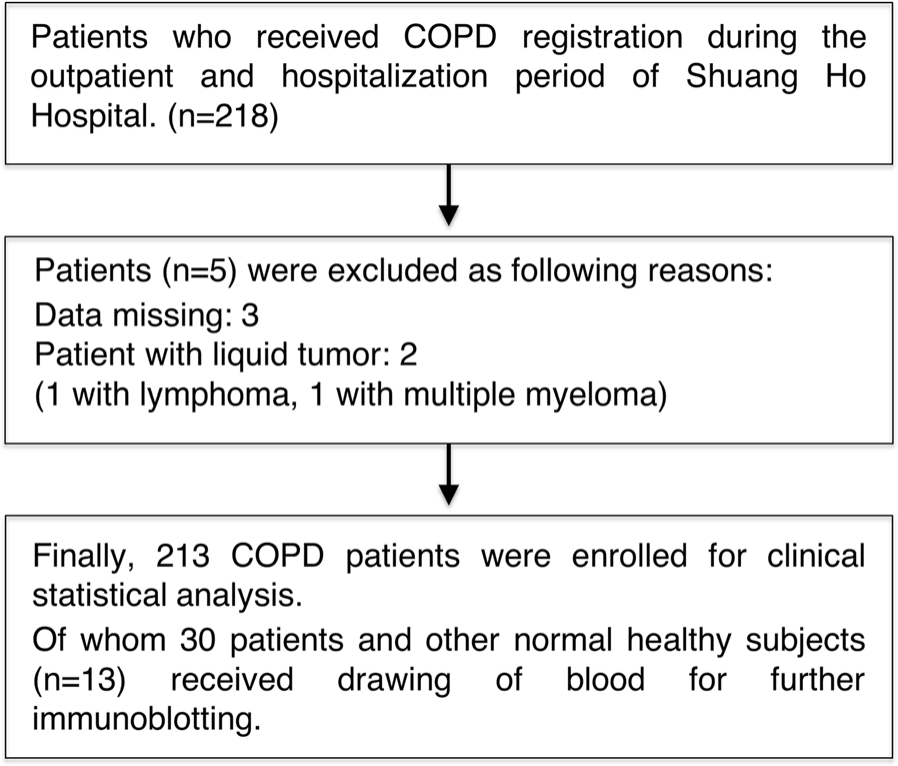
Patient enrollment flow chart

**Fig. 2:**
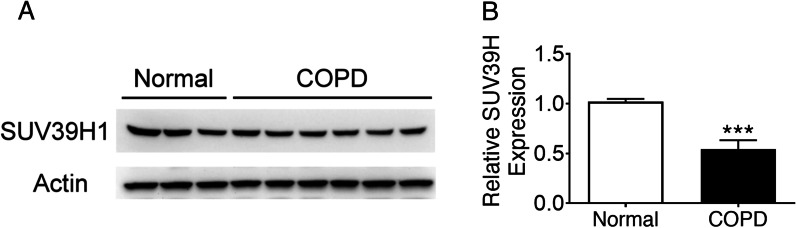
SUV39H1 levels are reduced in the peripheral blood mononuclear cells (PBMCs) of COPD patients. **a** The representative SUV39H1 expression in PBMC samples from normal control (n = 3) and COPD subjects (n = 7) was measured by immunoblotting. The levels of SUV39H1 expression were significantly reduced in the PBMCs from COPD patients compared with those from normal controls. Actin served as a loading control. **b** The densitometry values for SUV39H1 in normal (n = 13) or COPD PBMCs (n = 30) were quantified and normalized to the actin value. Relative expression values are expressed as the fold change over the normal control value. ***p < 0.001

The enrollment of study population is presented in Fig. 1. The levels of SUV39H1 protein in the peripheral blood mononuclear cells (PBMCs) of COPD patients (n = 30) and normal control (n = 13) were measured by Western blotting (Additional file 1: Figure 1). The characteristics of the study subjects with normal lungs or COPD evaluated with immunoblot assays are indicated in Additional file 2: Table 1. The lung function index was significantly decreased in the COPD patients compared with the normal controls, including smokers and non-smokers. Moreover, lung function was reduced in the more severe COPD patients compared with the mild COPD patients (GOLD Stage III/IV vs. I/II). As SUV39H1 controls genes encoding Th1 cytokines and non-specific inflammatory mediators [10], including IL-8, we tested whether low expression of SUV39H1 was associated with increased neutrophil counts. To this end, we divided the patients into low SUV39H1 expression (< 0.5-fold average of the normal subjects) and high SUV39H1 expression (≥ 0.5-fold average of the normal subjects) groups (Fig. 2). Additionally, characteristics of the COPD patients were compared between these two groups (Table 1). We found that the low SUV39H1 expression group had a significantly higher percentage of neutrophils in the blood (65.33% vs. 56.53%, *p* = 0.015). There was no difference in the total leukocyte count between the groups (7933/μL vs. 6773/μL, *p* = 0.110).

**Table 1:** Characteristics of COPD patients in low and high SUV39H1 expression groups

Variables	Low SUV Exp. (n = 15)	High SUV Exp. (n = 15)	*p* value
Male, n (%)	14 (93)	15 (100)	1
Age, years; median (IQR)	71 (64–77)	70 (62–74)	0.561
BMI; median (IQR)	24 (23.2–26.2)	22.7 (19.9–24.6)	0.141
*Cigarette smoking, n (%)*			0.413
Current smoker	5 (33)	8 (53)	
Ex-smoker	7 (47)	6 (40)	
Never smoker	3 (20)	1 (7)	
*Comorbidities, n (%)*			0.919
Heart failure	2 (13)	0 (0)	
Coronary artery disease	3 (20)	4 (27)	
Pulmonary hypertension	4 (27)	2 (13)	
Lung cancer	0 (0)	0 (0)	
Anxiety/depression	1 (7)	1 (7)	
Osteoporosis	0 (0)	1 (7)	
Malnutrition (BMI < 20)	2 (13)	4 (27)	
Diabetes mellitus	1 (7)	2 (13)	
OSA	1 (7)	1 (7)	
Anemia	2 (13)	2 (13)	
Lung fibrosis	1 (7)	0 (0)	
Charlson comorbidity Index score; median (IQR)	5 (3–5)	4 (4–6)	0.833
*Hemogram values; median (IQR)*			
Leukocyte count	7800 (6300–9200)	6600 (5950–7200)	0.110
Neutrophil, %	65 (60–71)	59 (52–61)	0.015*
Eosinophil, %	2 (1–3)	2 (2–4)	0.125
E/N Ratio, %	3.6 (1.7–4.7)	4.7 (3.7–7.1)	0.071
Pulmonary function tests; median (IQR)			
Post bronchodilator FEV1/FVC,%	62 (50–65)	60 (55–64)	0.868
Post bronchodilator FEV1, %	57 (35–67)	55 (40–73)	0.934
*COPD subtypes, n (%)*			0.966
Stage 1	2 (13)	2 (13)	
Stage 2	8 (54)	7 (47)	
Stage 3	2 (13)	3 (20)	
Stage 4	3 (20)	3 (20)	
ACO, n (%)	0 (0)	1 (6.7)	1
Exacerbations per year; median (IQR)	1 (0–1)	0 (0–1)	0.115

BMI, body mass index; E/N Ratio: Eosinophil/neutrophil count ratio; FEV1/FVC, first second of forced expiration/forced vital capacity; LAMA, long acting muscarinic antagonist; LABA, long-acting β2-agonists; ICS, inhaled corticosteroids; ACO, asthma-COPD overlap

^*^p < 0.05

Data are expressed as n, percentage, median and interquartile range (IQR, Q1–Q3) in bracket

Chi square comparison for Cigarette smoking, Comorbidities and COPD subtypes

Mann–Whitney U test for other variables

The low expression [fold change (FC) < 0.5] or high expression (FC ≥ 0.5) of SUV39H1 proteins in COPD patients relative to normal controls were indicated as “Low SUV Exp.” or “High SUV Exp.”, respectively

**Fig. 3:**
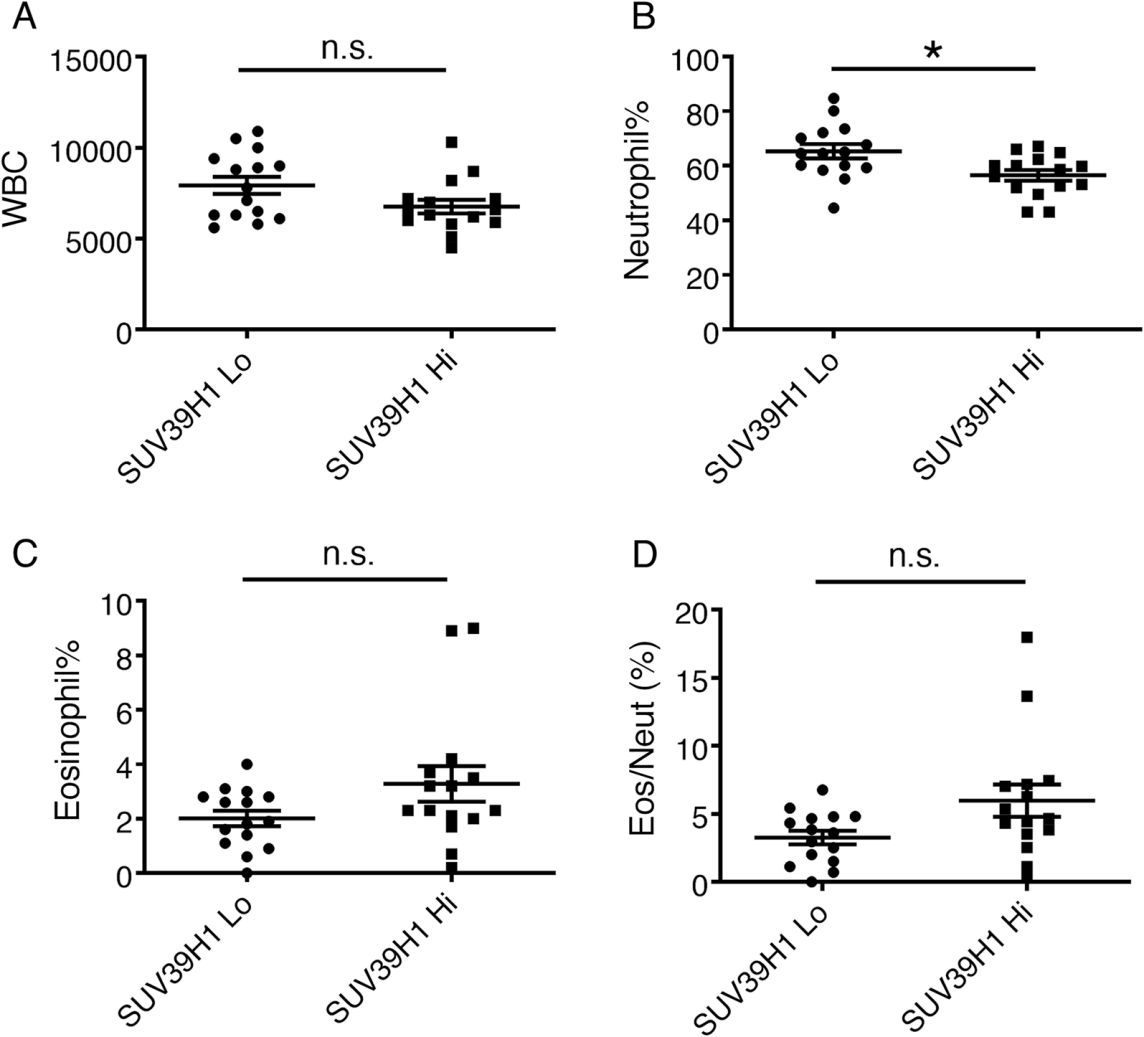
Blood neutrophil counts are increased in COPD patients with low SUV39H1 levels. **a** WBC counts were analysed in COPD patients with low SUV39H1 expression [fold change (FC) < 0.5, SUV39H1 Lo] or high SUV39H1 expression (FC ≥ 0.5, SUV39H1 Hi). However, there was no significant difference (*p* = 0.110). **b** Notably, the neutrophil percentage in COPD patients with low SUV39H1 expression was markedly higher than that in subjects with high SUV39H1 expression. *p = 0.015. **c** The eosinophil percentage in COPD patients with high SUV39H1 expression was increased compared with that in COPD patients with low SUV39H1 expression. However, there was no significant difference (*p* = 0.125). **d** Similarly, the ratios of eosinophils/neutrophils (Eos/Neuts) in COPD patients with high SUV39H1 expression were increased. However, the difference was not statistically significant (*p* = 0.071). n.s.: not significant

We also examined the levels of blood eosinophils, which are Th2 downstream effector cells. Although we found a trend towards an increase in the percentage of blood eosinophils in the high SUV39H1 group, the difference was not statistically significant (Fig. 3, 2.01% vs. 3.29%, *p* = 0.125). Interestingly, in contrast to the high SUV39H1 group, which had a wide variation in the percentage of blood eosinophils, the low SUV39H1 group had consistently low eosinophil percentages. The ratio of eosinophils/neutrophils showed a similar trend (3.3% vs. 6%, *p* = 0.071).

### Characteristics of the COPD cohort

Measuring SUV39H1 levels in PBMCs requires an adequate amount of blood; therefore, it was difficult to recruit more subjects to correlate SUV39H1 levels with additional clinical outcomes. We thus used an extended cohort to study blood cell counts instead. Medical records from 218 patients in our COPD registry cohort collected from March 2014 to December 2017 were reviewed; two patients were excluded for having liquid tumours, and three were excluded for missing data. Of the 213 enrolled patients, 112 (52.6%) were included in GOLD group A, 39 (18.3%) were included in group B, 21 (9.9%) were included in group C, and 41 (19.2%) were included in group D (Table 2). A total of 193 patients (90.6%) were male, the average age was 73.1 ± 8.4 years, and the average BMI was 23.57 ± 4.11. Eighty patients (37.5%) were current smokers, 113 patients (53.1%) were ex-smokers, and 20 patients (9.4%) were never smokers. Nineteen patients (8.9%) had a history of asthma or met the diagnostic criteria of ACO, which was defined in the joint project of GOLD and GINA in 2015.

**Table 2:** Characteristics of patients with COPD

Variables	COPD patients (n = 213)
Male, n (%)	193 (90.6)
Age, years (mean ± SD)	73.1 ± 8.4
BMI (mean ± SD)	23.57 ± 4.11
*Cigarette smoking, n (%)*	
Current smoker	80 (37.5)
Ex-smoker	113 (53.1)
Never smoker	20 (9.4)
*Comorbidities, n (%)*	
Heart failure	17 (7.98)
Coronary artery disease	54 (25.35)
Pulmonary hypertension	28 (13.15)
Lung cancer	3 (1.4)
Anxiety/depression	9 (4.2)
Osteoporosis	12 (5.63)
Malnutrition (BMI < 20)	38 (17.84)
Diabetes mellitus	34 (15.96)
OSA	8 (3.76)
Normocytic anemia	26 (12.2)
Lung fibrosis	14 (6.57)
Charlson comorbidity index score; median (IQR)	5 (4–6)
*Hemogram values; median (IQR)*	
Leukocyte count	7800 (6500–9400)
Neutrophil, %	62.9 (54.3–71.1)
Eosinophil, %	2.2 (1.0–4.0)
*Pulmonary function tests; median (IQR)*	
Post bronchodilator FEV1/FVC, %	56 (40.2–72.2)
Post bronchodilator FEV1, %	56 (47–63.4)
*COPD subtypes, n (%)*	
GOLD I	29 (13.6)
GOLD II	107 (50.2)
GOLD III	52 (24.4)
GOLD IV	25 (11.8)
Group A	112 (52.6)
Group B	39 (18.3)
Group C	21 (9.9)
Group D	41 (19.2)
*Treatment, n (%)*	
LABA + LAMA + ICS	66 (30.99)
LABA + LAMA	76 (35.68)
LABA + ICS	18 (8.45)
LAMA + ICS	1 (0.47)
LAMA only	36 (16.9)
LABA only	12 (5.63)
No inhaler treatment	4 (1.88)
ACO, n (%)	19 (8.92)
Exacerbations per year; median (IQR)	1 (0–1)

BMI, body mass index; FEV1/FVC, first second of forced expiration/ forced vital capacity; LAMA, long acting muscarinic antagonist; LABA, long-acting β2-agonists; ICS, inhaled corticosteroids; ACO, asthma-COPD overlap; interquartile range (IQR, Q1–Q3)

The most common COPD-related comorbidities were coronary artery disease (n = 54, 25.35%), malnutrition (n = 38, 17.8%), diabetes mellitus (n = 34, 16.0%), pulmonary hypertension (n = 28, 13.2%), normocytic anaemia (n = 26, 12.2%), heart failure (n = 17, 8.0%), lung fibrosis (n = 14, 6.6%), osteoporosis (n = 12, 5.6%), anxiety/depression (n = 9, 4.2%), obstructive sleep apnoea (n = 8, 3.8%), and lung cancer (n = 3, 1.4%). The Charlson Comorbidity Index score was 1.1 in average.

Regarding medications, 76 patients (35.7%) received dual bronchodilators (LABA + LAMA), 66 (31.0%) received triple therapy (LABA + LAMA + ICS), 36 (16.9%) received LAMA only, 18 (8.4%) received LABA + ICS, 12 (5.6%) received LABA only, 1 (0.5%) received LAMA + ICS, and 4 (1.9%) did not receive any inhaled treatment.

### Neutrophilia was correlated with COPD comorbidities but not the frequency of moderate to severe exacerbations

**Fig. 4:**
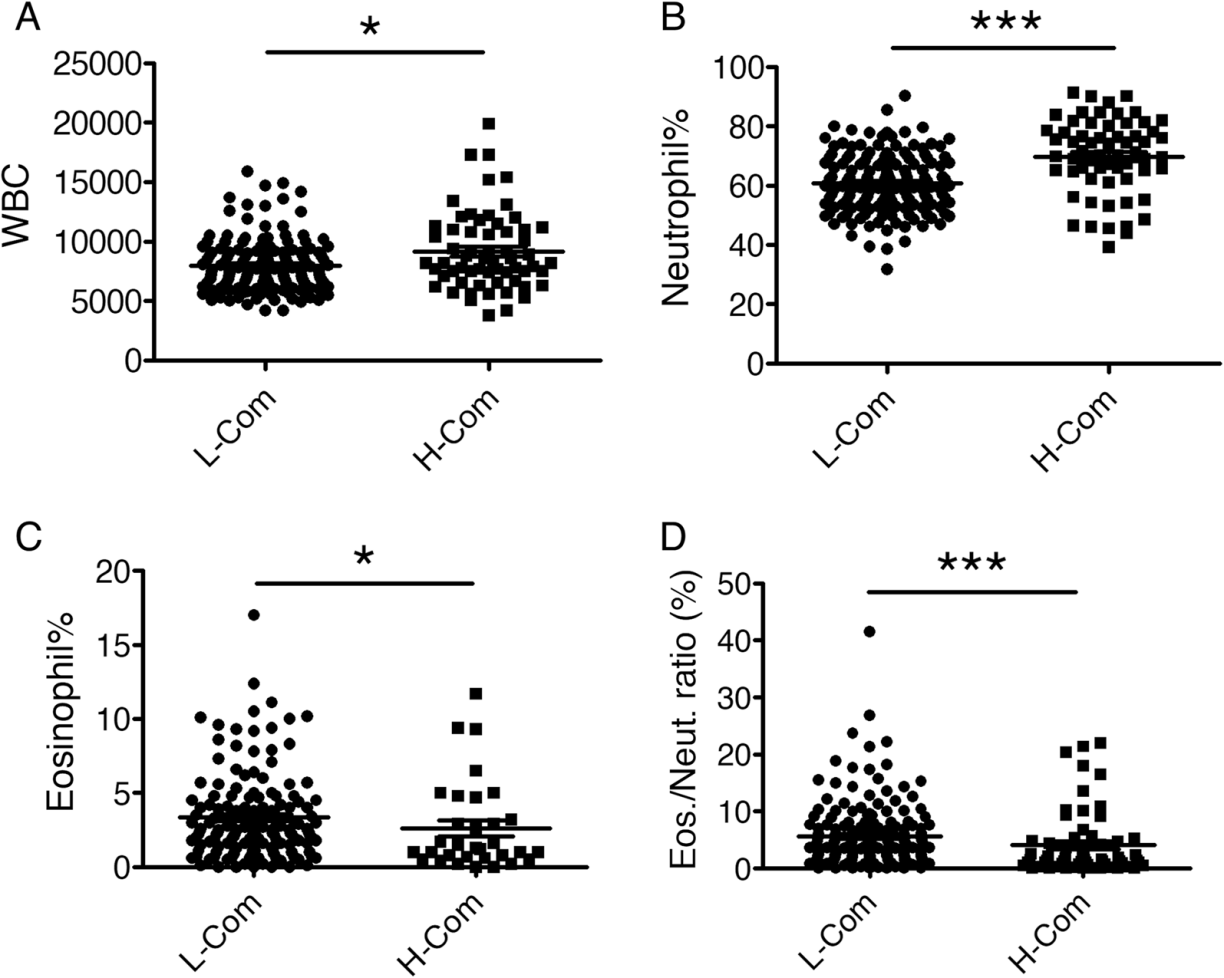
Blood neutrophil counts are increased in COPD patients with high comorbidities. WBC and differential counts in COPD patients with 0–1 comorbidities (L-Com) were compared with those in COPD patients with ≥ 2 comorbidities (H-Com) by statistical analysis. **a** Peripheral blood WBC counts were increased in COPD subjects with high comorbidities (H-Com) compared with those with low comorbidities (L-Com). *p = 0.012. **b** Moreover, neutrophil counts were greater in COPD subjects with H-Com than in those with L-Com. ***p < 0.001. **c** However, peripheral blood eosinophil counts were reduced in COPD subjects with H-Com. *p = 0.037. **d** The ratio of peripheral blood eosinophils/neutrophils was decreased in COPD subjects with H-Com. ***p < 0.001

**Fig. 5:**
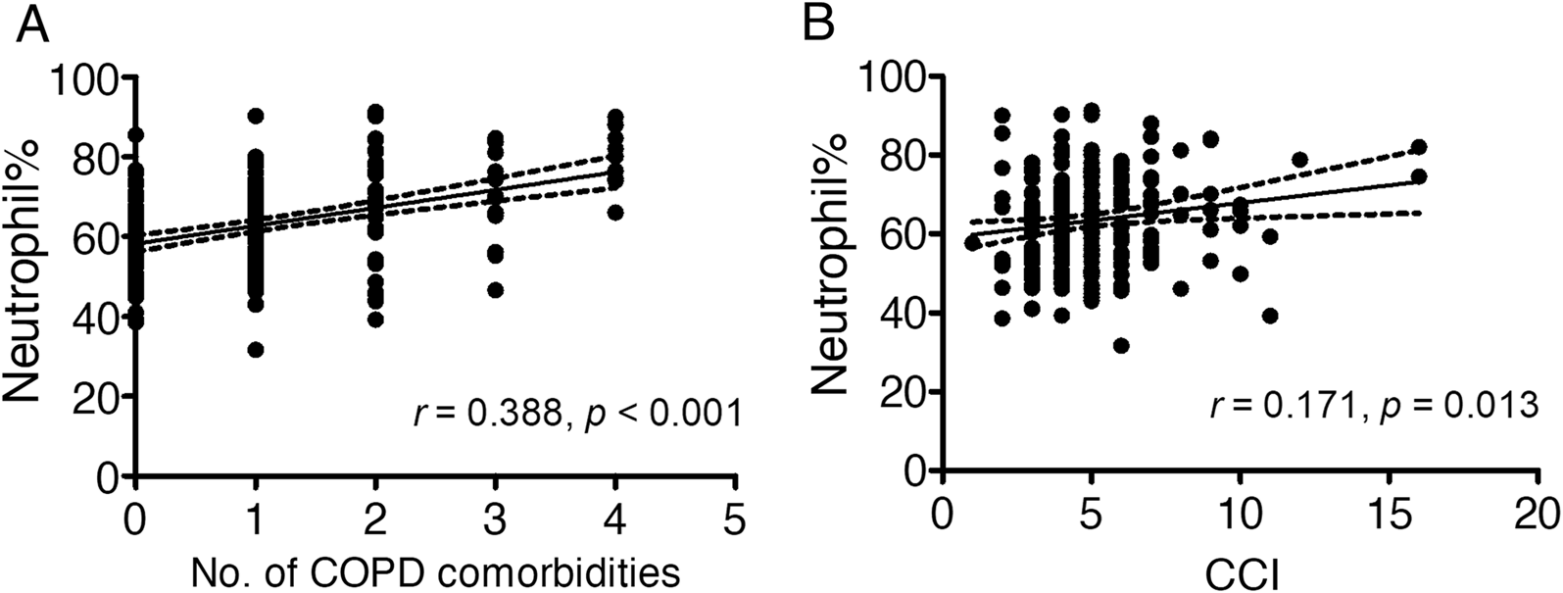
Correlations between the percentage of neutrophils and number of COPD comorbidities or CCI. **a** The correlation between the peripheral blood neutrophil count and number of COPD comorbidities was analysed by Spearman’s rank correlation analysis. Spearman’s coefficient r = 0.388, *p* < 0.001. **b** The association between the peripheral blood neutrophil count and CCI was assessed. Spearman’s r = 0.171, *p* = 0.013

To identify the associations of systemic inflammation with comorbidities, we grouped patients with COPD into two groups according to the number of comorbidities (low: 0–1 comorbidities; high: ≥ 2 comorbidities) (Table 3). We found that the high comorbidity group had a lower BMI (22.78 ± 5.41 vs 23.9 ± 3.41, *p* = 0.026), a smaller percentage of Group A COPD patients (35.5% vs. 59.6%, *p* = 0.0014), higher percentages of GOLD 4 (22.6% vs. 7.3%, *p* = 0.002) and Group D COPD patients (32.3% vs. 13.9%, *p* = 0.002), higher total leukocyte counts (9,187/μL vs. 7,983/μL, *p* = 0.012), a higher neutrophil percentage (69.8% vs. 60.7%, *p* < 0.001), a lower eosinophil percentage (2.3% vs. 3.3%, *p* = 0.037), and a lower eosinophil/neutrophil ratio (4.1% vs. 6.03%, *p* < 0.001) (Fig. 4). The neutrophil ratio seemed to have the most significant difference. Next, we examined whether specific comorbidities related to neutrophilia. However, post hoc analysis using Dunn’s multiple comparison tests did not show statistical significance for any single comorbidity (Additional file 1: Figure 2). For the comparison of comorbidities, the average number of comorbidities in the high comorbidity group was 2.58 (vs. 0.55 in the low comorbidity group, *p* < 0.001). The high comorbidity group had significantly more incidences of all comorbidities except lung cancer (3.2% vs. 0.7%, *p* = 0.149). Further analyses revealed that the neutrophil percentage was more positively correlated with the number of comorbidities (Spearman's rank correlation coefficient r = 0.388, *p* < 0.001) than the Charlson Comorbidity Index (CCI) scores (Spearman’s r = 0.171, *p* = 0.013) (Fig. 5).

**Table 3:** Characteristics of COPD patients in low and high comorbidity groups

Variables	Low cormobidity (n = 151)	High cormobidity (n = 62)	*p* value
Male, n (%)	138 (91.4)	55 (88.7)	0.542
Age, years (mean ± SD)	71.7 ± 8.6	75 ± 8.1	0.115
BMI (mean ± SD)	23.9 ± 3.41	22.78 ± 5.41	0.026
*Cigarette smoking, n (%)*			0.804
Current smoker	58 (38.4)	22 (35.5)	
Ex-smoker	80 (53.0)	33 (53.2)	
Never smoker	13 (8.6)	7 (11.3)	
No. of comorbidities, median (IQR)	1 (0–1)	2 (2–3)	< 0.0001***^a^
*Comorbidities, n (%)*			
Heart failure	6 (4.0)	11 (17.7)	< 0.001***^a^
Coronary artery disease	21 (13.9)	33 (53.2)	< 0.001***
Pulmonary hypertension	9 (6.0)	19 (30.6)	< 0.001***
Lung cancer	1 (0.7)	2 (3.2)	0.149
Anxiety/depression	3 (2.0)	6 (9.7)	0.011*
Osteoporosis	5 (3.3)	7 (11.3)	0.022*
Malnutrition (BMI < 20)	15 (9.9)	23 (37.1)	< 0.0001***
Diabetes mellitus	12 (7.9)	22 (35.5)	< 0.0001***
OSA	1 (0.7)	7 (11.3)	0.0002**
Anemia	4 (2.6)	22 (35.5)	< 0.0001***
Lung fibrosis	6 (4.0)	8 (12.9)	0.017*
Charlson comorbidity index score, median (IQR)	4 (3–5)	6 (5–7)	< 0.0001***
*Hemogram values, median (IQR)*			
Leukocyte count	7500 (6300–9100)	8300 (7175–11,000)	0.0121*
Neutrophil, %	60 (52.9–68.2)	70.6 (64.9–78.4)	< 0.0001***
Eosinophil, %	2.5 (1.4–4.3)	1.0 (0.5–3)	0.0005***
*Pulmonary function tests, median (IQR)*			
Post bronchodilator FEV1/FVC%	58 (49.1–64.2)	51.8 (42.6–59.8)	0.011*
Post bronchodilator FEV1%	60.5 (43–74.4)	51.8 (32.8–63.8)	0.004**
*COPD subtypes, n (%)*			0.012*
COLD I	22 (14.6)	7 (11.3)	
GOLD II	82 (54.3)	25 (40.3)	
GOLD III	36 (23.8)	16 (25.8)	
GOLD IV	11 (7.3)	14 (22.6)	
*COPD subtypes, n (%)*			0.0038**
Group A	90 (59.6)	22 (35.5)	
Group B	25 (16.6)	14 (22.6)	
Group C	15 (9.9)	6 (9.7)	
Group D	21 (13.9)	20 (32.3)	
*Treatment, n (%)*			
LABA + LAMA + ICS	44 (29.1)	22 (35.5)	0.363
LABA + LAMA	53 (35.1)	23 (37.1)	0.782
LABA + ICS	14 (9.3)	4 (6.4)	0.502
LAMA + ICS	1 (0.7)	0 (0)	0.521
LAMA only	24 (15.9)	12 (19.4)	0.540
LABA only	11 (7.3)	1 (1.6)	0.103
No inhaler treatment	4 (2.6)	0 (0)	0.196
ACO, n (%)	16 (10.6)	3 (4.8)	0.181
Exacerbations per year, median (IQR)	0 (0–1)	1 (0–2)	0.005**

Data are expressed as n, mean ± SD (the data of age and BMI are normally distributed), or percentage, median and interquartile range (IQR, Q1–Q3) in bracket

Chi square comparison for Cigarette smoking, Comorbidities and COPD subtypes

Mann–Whitney U test for other variables

The low comorbidity group (0 and 1 comorbidities) and high comorbidity group (≥ 2 comorbidities) of COPD patients were indicated as “Low Cormobidity” and “High Cormobidity”, respectively

BMI, body mass index; FEV1/FVC, first second of forced expiration/ forced vital capacity; LAMA, long acting muscarinic antagonist; LABA, long-acting β2-agonists; ICS, inhaled corticosteroids; ACO, asthma-COPD overlap

*p < 0.05, **p < 0.01, *** *p* < 0.001

**Fig. 6:**
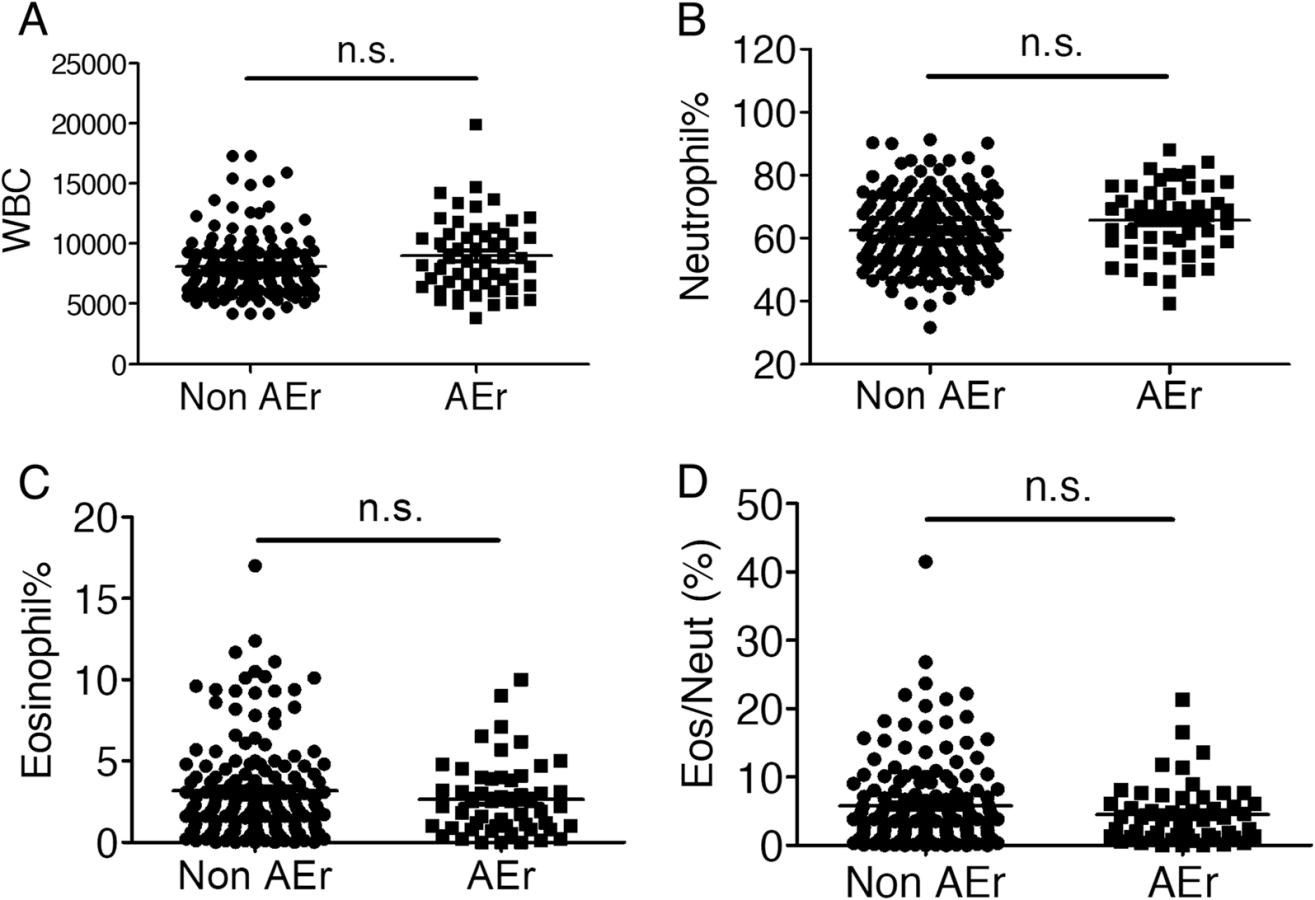
Blood neutrophil and eosinophil counts are not significantly related to moderate to severe COPD exacerbations. WBC and differential counts in COPD patients with 0–1 moderate to severe exacerbations in the past 1 year (non-AEr) were compared with those in patients with ≥ 2 moderate to severe exacerbations in the past 1 year (AEr) by statistical analysis. **a** The peripheral blood WBC counts were similar in COPD subjects with few exacerbations annually (non-AEr) and those with frequent exacerbations (AEr). *p* = 0.078. **b** Neutrophil counts were also similar in both groups. *p* = 0.061. **c** Eosinophil counts were not different between the groups. *p* = 0.570 (**d**) The ratio of peripheral blood eosinophils/neutrophils was similar in both groups. *p* = 0.397. n.s.: not significant

In this study, we found that the high comorbidity group also had more moderate to severe exacerbations per year (1.5 vs. 0.9, *p* = 0.005). To confirm that the actual role of neutrophils is related to the number of comorbidities or frequency of exacerbations, we tested the associations of blood cell counts with moderate to severe exacerbations of COPD (Fig. 6) by dividing the patients into two groups: patients with non-frequent exacerbations (annual exacerbation rates of 0–1 exacerbations/year) and patients with frequent exacerbations (more than 1 exacerbation/year). There were no significant differences between these two groups in the total white blood cell count (*p* = 0.078), neutrophil percentage (*p* = 0.061), eosinophil percentage (*p* = 0.570), or eosinophil/neutrophil ratio (*p* = 0.397).

### Neutrophilia is increased in groups with high COPD-related comorbidities

**Fig. 7:**
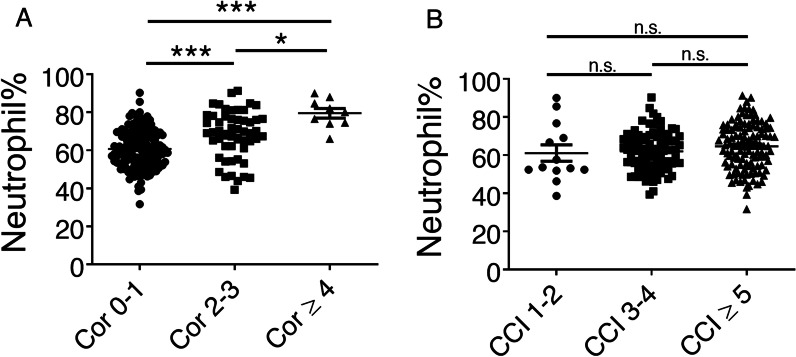
Neutrophilia is increased in groups with high COPD-related comorbidities. The neutrophil percentage of the peripheral blood in COPD patients with different numbers of comorbidities and CCI scores were analysed. **a** The neutrophil percentage was significantly increased in the high COPD-related comorbidity groups (Cor 2–3 and Cor ≥ 4) compared with the low comorbidity group (Cor 0–1). This comparison was analysed by one-way ANOVA, with post hoc analysis by Dunn’s multiple comparison test. The post hoc analysis in each subgroup comparison revealed the following significant differences: Cor 0–1 versus Cor 2–3, ***p < 0.0001; Cor 0–1 versus Cor ≥ 4, ***p < 0.0001; and Cor 2–3 versus Cor ≥ 4, *p < 0.05. **b** The neutrophil percentages in the high CCI subgroups (CCI 3–4, ≥ 5) were not significantly different from that in the low CCI subgroup (CCI 1–2). The post hoc analysis comparing all subgroups also revealed no significant differences. n.s.: not significant

The number of comorbidities in our study was divided into few comorbidities (0–1) and high comorbidities (more than 2), which we showed in this study to be equivalent to the results for the CCI, and a relatively high correlation with COPD-related comorbidities was revealed. Thus, the neutrophil percentage in the peripheral blood of COPD patients with different numbers of comorbidities and CCI were further examined. Our data showed that the neutrophil percentage was significantly increased in the high COPD-related comorbidity groups (Cor 2–3 and Cor ≥ 4) compared with the low comorbidity group (Cor 0–1) (Fig. 7a). The neutrophil percentages in the high CCI subgroups (CCI 3–4 and ≥ 5) were not significantly increased compared with that in the low CCI subgroup (Fig. 7b). Our results are consistent with previous findings [1]. Collectively, our results showed that the neutrophil percentage in COPD patients was markedly increased in those patients with a high number of comorbidities but not in those patients with a high CCI score (Fig. 7).

### Reduced SUV39H1 expression was associated with COPD comorbidities

**Fig. 8:**
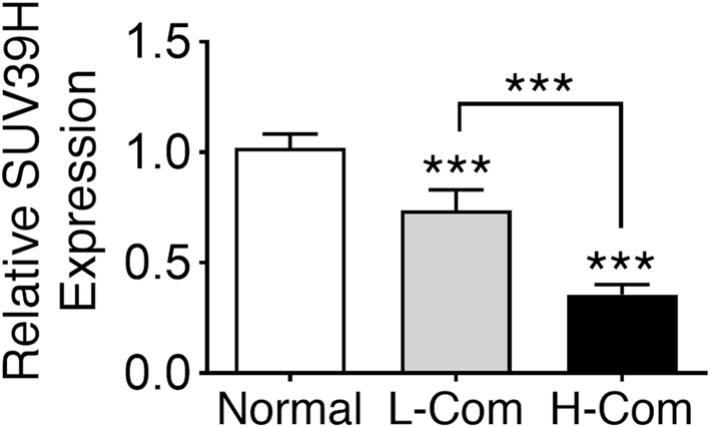
The levels of SUV39H1 are significantly reduced in COPD patients with high comorbidities. The levels of SUV39H1 protein expression in PBMCs from normal control (n = 13) and COPD subjects (L-Com: ≤ 1 comorbidity, n = 15 vs. H-Com: ≥ 2 comorbidities, n = 15) were measured by immunoblotting. The protein intensity values for SUV39H1 were normalized to the actin value and are expressed as the fold change over the control value. **p < 0.01

As reduced SUV39H1 expression was related to neutrophilia, we next asked whether SUV39H1 is associated with comorbidities. By reanalysing the densitometry Western blotting data, we found a modest reduction in the SUV39H1 level in patients with low comorbidities (Fig. 8). Moreover, lower levels of SUV39H1 in PBMCs were observed in COPD patients with high comorbidities than in those with low comorbidities. These data together suggest that impaired SUV39H1 expression leads to neutrophilia and thus comorbidities.

**Fig. 9:**
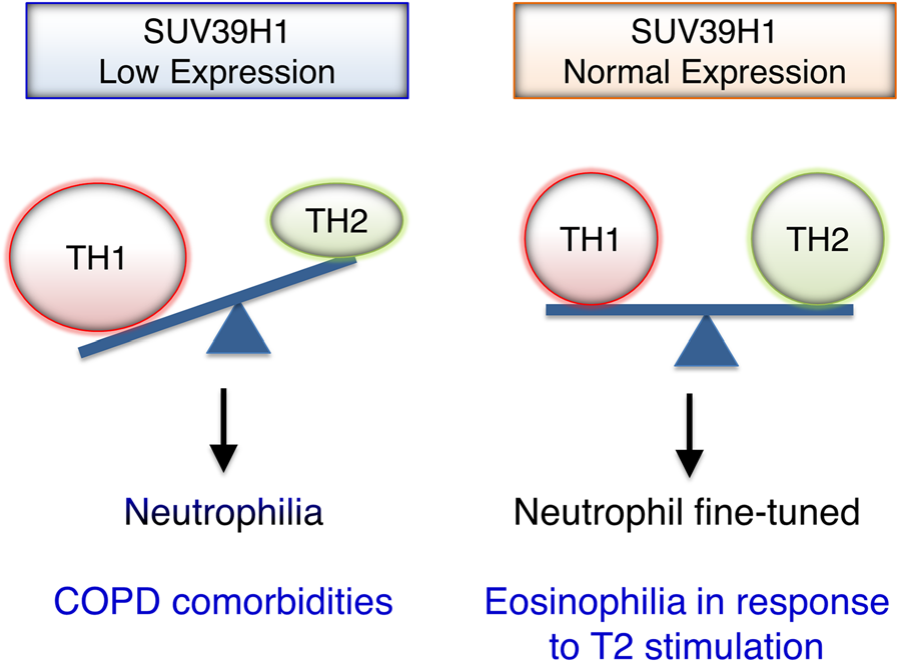
Working model of SUV39H1 in the Th1/Th2 balance and its impact on inflammation in COPD. Normal SUV39H1 expression controls Th1 genes and maintains the balance between Th1 and Th2 responses. Eosinophilic inflammation can occur when Th2 stimulation is present. In more severe COPD, the depleted expression of SUV39H1 skews immunity towards Th1 inflammation, suppressing any possible eosinophilic inflammation and causing more dominant neutrophilia and COPD comorbidities

Collectively, reduced SUV39H1 expression in COPD patients is associated with neutrophilia and thus comorbidities. We suggest that preserving the expression of SUV39H1 may control the Th1 response and maintain the balance between Th1 and Th2 responses (Fig. 9). Patients in this condition may have milder or eosinophilic inflammation once they also have asthma or other Th2-related conditions. This inference might indicate relatively good outcomes for eosinophilic COPD. In more severe COPD patients, the nearly depleted expression of SUV39H1 skews Th1 polarization, causing more dominant neutrophilia and COPD comorbidities.

## Discussion

We discovered that the levels of SUV39H1 were reduced and associated with the severity of COPD in previous work [10]. In the present study, we confirmed the reduction in the SUV39H1 level in patients with COPD by using a previously defined cohort (Fig. 2) and extended our previous finding to show that reduced SUV39H1 expression in PBMCs was not only associated with systemic inflammation but also linked to comorbidities. In this study, our results indicated that neutrophilia was associated with comorbidities in COPD patients. By contrast, eosinophilia was associated with fewer comorbidities, with patients exhibiting relatively normal SUV39H1 levels. The link between SUV39H1 and comorbidities is supported by a report by Yang et al*.* [14]. In their study, genetic deletion or pharmaceutical inhibition of SUV39H worsened cardiac injury following myocardial infarction in mice.

Our study extends our knowledge on the clinical relevance of neutrophilia in COPD—neutrophilia is associated with comorbidities. Until the present study, the roles of neutrophils in COPD were known to be related to disease pathophysiology and progression in the lungs, including chronic expectoration [15], a rapid decline in FEV1 [16, 17], and the development of emphysema [18]. In the ECLIPSE study, both neutrophil and white cell counts were very weak risk factors for acute exacerbation [19]. In our study, neither of those blood cell counts was significantly associated with exacerbation. This is very likely due to the too small number of patients compared with the sample size in the ECLIPSE study, which was sufficient to show small differences. It is worth confirming the predictive value of neutrophilia for comorbidities in a large-scale setting. The present study also provided evidence supporting the relationship between systemic inflammation and comorbidities.

In the present study, we analysed 11 comorbidities based on the report of Barnes et al*.* published in 2009 [1]. However, our results revealed no statistical significance for any single comorbidity correlated with neutrophilia. The Charlson Comorbidity Index (CCI) is a common scoring system for the prediction of mortality and prognosis in many diseases, including COPD [20]. In our study, the correlation of neutrophilia with the CCI was less significant than the correlations with the numbers of the 11 comorbidities. These results suggested that the cause of this difference relied on the lack of malnutrition, pulmonary hypertension, and normocytic anaemia in the list of CCI. Thus, numbers of COPD-specific comorbidities rather than CCI scores might be an ideal tool for reflecting the systemic inflammation in COPD.

The negative correlation of SUV39H1 expression with neutrophilia supports the participation of the SUV39H1-H3K9me3-HP1α pathway in silencing genes encoding nonspecific inflammation in COPD [10]. An increased number of neutrophils is evident in all stages of COPD, and these cells were shown to move faster but with reduced migratory accuracy in the presence of IL-8, growth-related oncogene alpha, and formyl-methionyl-leucyl-phenylalanine [21], even after cigarette cessation [22]. In our observations, most patients had downregulated SUV39H1 expression, which is expected to produce more IL-8 and thus more neutrophils. We thus infer that low SUV39H1 expression/neutrophilia is a typical phenotype of COPD. Under the antagonistic effect of high Th1 cytokine levels, the Th2 response cannot be easily mounted. In other words, eosinophilia will not be common in patients with COPD, particularly in those with low SUV39H1 expression levels.

Eosinophilia was associated with low comorbidities in the present study. While eosinophilia has either no relationship or a positive relationship with exacerbation outcomes [23], it seems to be associated with better outcomes in terms of lung function decline and mortality [24]. Thus, our observation is consistent with previous observations. We found a trend towards increased blood eosinophil counts in patients with relatively normal SUV39H1 expression, but the difference was not significant. We infer that relatively intact SUV39H1 expression ensures the initiation of eosinophilic inflammation in patients with Th2 skewing, which was proved in a mouse model of asthma, reported suppressed SUV39H1 to skew T cell responses towards the Th1 response, whereas functioning SUV39H1 ensured Th2 lineage stability [9].

Regarding the potential effect on ACO patients in this study, we have some reasons to enroll them. First, a previous report showed that the prevalence of ACO varied widely from 12.6 to 55.7% in patients with COPD [25]. The proportion was 8.92% in our study population; thus, we cannot exclude it. Second, we found no significant influences of the haemogram profile or number of comorbidities on these ACO patients. As shown in Table 3, there was also no significant difference in the proportion of ACO patients between the low and high comorbidity groups.

## Conclusion

In this study we reported the blood neutrophil counts are associated with comorbidities in COPD patients. Impaired SUV39H1 expression in PBMCs from COPD patients are correlated with neutrophilic inflammation and comorbidities.

## Supplementary Information

**Additional file 1**: Figure 1. The levels SUV39H1 proteins are reduced in the peripheral blood mononuclear cells (PBMCs) of COPD patients. Figure 2. The proportion of Neutrophilia in COPD patients compared with all comorbidity groups.

**Additional file 2**: Table 1. Characteristics of the study subjects with normal or COPD for immunoblot assays.

## Abbreviations

COPD: Chronic obstructive pulmonary disease
Th1: T helper cell type 1
Th2: T helper cell type 2
PBMCs: Peripheral blood mononuclear cells
SUV39H1: Suppressor of variegation 3–9 homolog 1
WBC: White blood cells
CCI: Charlson comorbidity index
FEV1: Forced expiratory volume in 1s
FVC: Forced vital capacity

## Declarations

### Availability of data and materials

The dataset used and/or analyzed during the current study are available from the corresponding author on reasonable request.

## References

[1] Barnes PJ, Celli BR (2009). Systemic manifestations and comorbidities of COPD. Eur Respir J.

[2] Deniz S, Sengul A, Aydemir Y, Celdir Emre J, Ozhan MH (2016). Clinical factors and comorbidities affecting the cost of hospital-treated COPD. Int J Chron Obstruct Pulmon Dis.

[3] Currie GP, Lipworth BJ (2016). Inhaled treatment for chronic obstructive pulmonary disease: what's new and how does it fit?. QJM.

[4] Maqsood U, Ho TN, Palmer K, Eccles FJ, Munavvar M, Wang R (2019). Once daily long-acting beta2-agonists and long-acting muscarinic antagonists in a combined inhaler versus placebo for chronic obstructive pulmonary disease. Cochrane Database Syst Rev.

[5] Kostikas K, Brindicci C, Patalano F (2018). Blood eosinophils as biomarkers to drive treatment choices in asthma and COPD. Curr Drug Targets.

[6] Harries TH, Rowland V, Corrigan CJ, Marshall IJ, McDonnell L, Prasad V (2020). Blood eosinophil count, a marker of inhaled corticosteroid effectiveness in preventing COPD exacerbations in post-hoc RCT and observational studies: systematic review and meta-analysis. Respir Res.

[7] Narendra DK, Hanania NA (2019). Targeting IL-5 in COPD. Int J Chron Obstruct Pulmon Dis.

[8] Suzuki M, Makita H, Konno S, Shimizu K, Kimura H, Kimura H (2016). Asthma-like features and clinical course of chronic obstructive pulmonary disease. An analysis from the Hokkaido COPD cohort study. Am J Respir Crit Care Med.

[9] Allan RS, Zueva E, Cammas F, Schreiber HA, Masson V, Belz GT (2012). An epigenetic silencing pathway controlling T helper 2 cell lineage commitment. Nature.

[10] Chen TT, Wu SM, Ho SC, Chuang HC, Liu CY, Chan YF (2017). SUV39H1 reduction is implicated in abnormal inflammation in COPD. Sci Rep.

[11] Vestbo J, Hurd SS, Agusti AG, Jones PW, Vogelmeier C, Anzueto A (2013). Global strategy for the diagnosis, management, and prevention of chronic obstructive pulmonary disease: GOLD executive summary. Am J Respir Crit Care Med.

[12] Charlson ME, Pompei P, Ales KL, MacKenzie CR (1987). A new method of classifying prognostic comorbidity in longitudinal studies: development and validation. J Chronic Dis.

[13] Lee KY, Ho SC, Chan YF, Wang CH, Huang CD, Liu WT (2012). Reduced nuclear factor-kappaB repressing factor: a link toward systemic inflammation in COPD. Eur Respir J.

[14] Yang G, Weng X, Zhao Y, Zhang X, Hu Y, Dai X (2017). The histone H3K9 methyltransferase SUV39H links SIRT1 repression to myocardial infarction. Nat Commun.

[15] Kim S, Nadel JA (2004). Role of neutrophils in mucus hypersecretion in COPD and implications for therapy. Treat Respir Med.

[16] Stanescu D, Sanna A, Veriter C, Kostianev S, Calcagni PG, Fabbri LM (1996). Airways obstruction, chronic expectoration, and rapid decline of FEV1 in smokers are associated with increased levels of sputum neutrophils. Thorax.

[17] Donaldson GC, Seemungal TA, Patel IS, Bhowmik A, Wilkinson TM, Hurst JR (2005). Airway and systemic inflammation and decline in lung function in patients with COPD. Chest.

[18] Parr DG, White AJ, Bayley DL, Guest PJ, Stockley RA (2006). Inflammation in sputum relates to progression of disease in subjects with COPD: a prospective descriptive study. Respir Res.

[19] Faner R, Tal-Singer R, Riley JH, Celli B, Vestbo J, MacNee W (2014). Lessons from ECLIPSE: a review of COPD biomarkers. Thorax.

[20] Figueira-Goncalves JM, Golpe R, Garcia-Bello MA, Garcia-Talavera I, Castro-Anon O (2019). Comparison of the prognostic capability of two comorbidity indices in patients with chronic obstructive pulmonary disease, in real-life clinical practice. Clin Respir J.

[21] Sapey E, Stockley JA, Greenwood H, Ahmad A, Bayley D, Lord JM (2011). Behavioral and structural differences in migrating peripheral neutrophils from patients with chronic obstructive pulmonary disease. Am J Respir Crit Care Med.

[22] Louhelainen N, Rytila P, Haahtela T, Kinnula VL, Djukanovic R (2009). Persistence of oxidant and protease burden in the airways after smoking cessation. BMC Pulm Med.

[23] Kim VL, Coombs NA, Staples KJ, Ostridge KK, Williams NP, Wootton SA, et al. Impact and associations of eosinophilic inflammation in COPD: analysis of the AERIS cohort. Eur Respir J. 2017;50(4). 10.1183/13993003.00853-2017 29025891

[24] Ho J, He W, Chan MTV, Tse G, Liu T, Wong SH (2017). Eosinophilia and clinical outcome of chronic obstructive pulmonary disease: a meta-analysis. Sci Rep.

[25] Hosseini M, Almasi-Hashiani A, Sepidarkish M, Maroufizadeh S (2019). Global prevalence of asthma-COPD overlap (ACO) in the general population: a systematic review and meta-analysis. Respir Res.

