# Supplementary material for: Suppressor of variegation 3–9 homologue 1 impairment and neutrophil-skewed systemic inflammation are associated with comorbidities in COPD
Source: BMC Pulm Med. 2021 Oct 2;21:276. doi: 10.1186/s12890-021-01628-x (PMC8487160; doi:10.1186/s12890-021-01628-x)
Supplement: Supplementary file 1 — Additional file 1: Figure 1. The levels SUV39H1 proteins are reduced in the peripheral blood mononuclear cells (PBMCs) of COPD patients. Figure 2. The proportion of Neutrophilia in COPD patients compared with all comorbidity groups. [file 12890_2021_1628_MOESM1_ESM.docx]

**
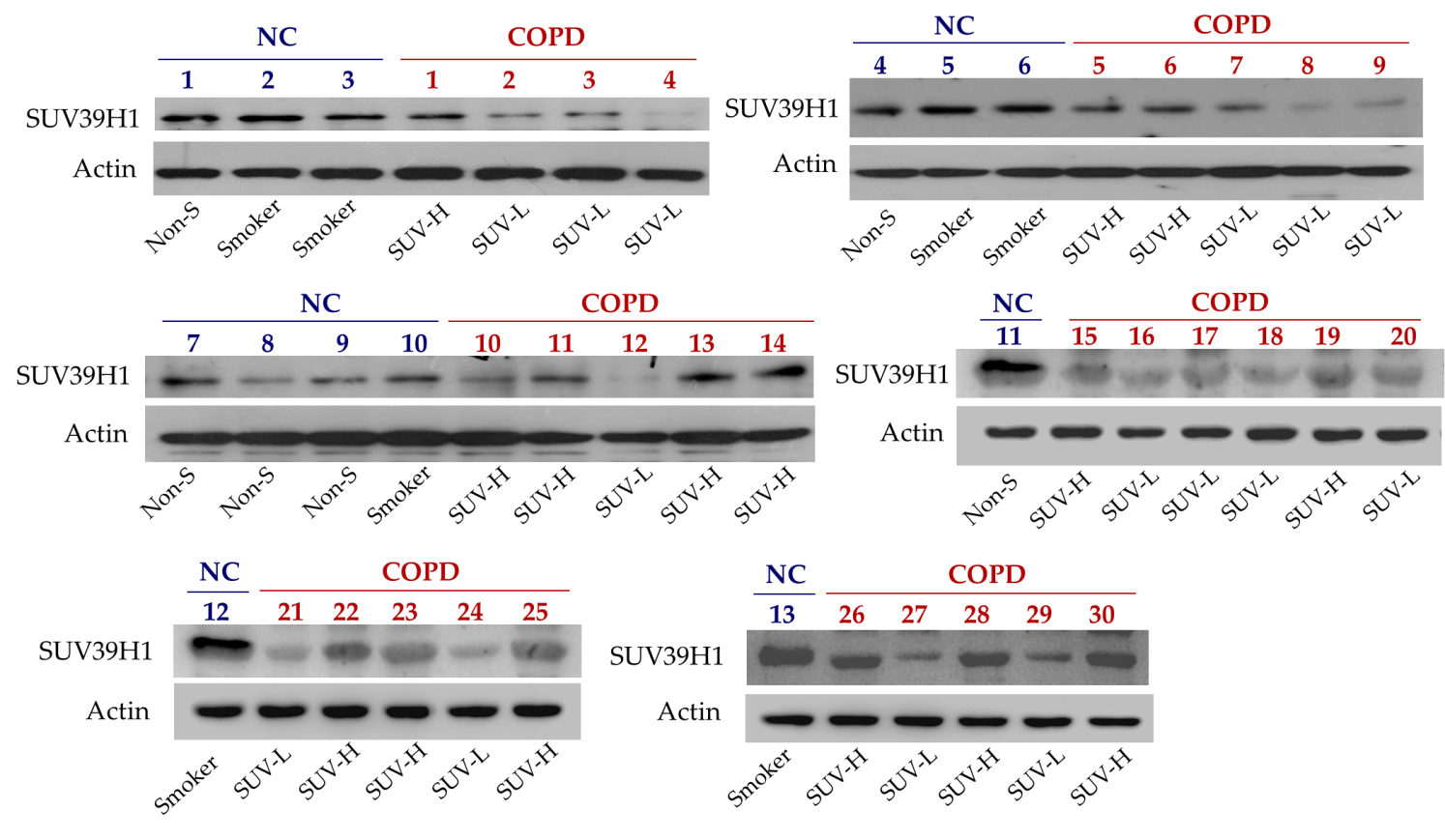
**

**Supplementary Figure 1. The levels SUV39H1 proteins are reduced in the peripheral blood mononuclear cells (PBMCs) of COPD patients.** The representative SUV39H1 expression in PBMC samples from normal control (including non-smokers and smokers (NC, n=13)) and COPD subjects (n=30) was measured by Western blotting. The densitometry values for SUV39H1 in normal or COPD PBMCs were quantified and normalized to the actin value. The low expression [fold change (FC) < 0.5] or high expression (FC ≥ 0.5) of SUV39H1 proteins in COPD patients were labeled as SUV-L or SUV-H, respectively.

**Supplementary Figure 2. The proportion of Neutrophilia in COPD patients compared with all comorbidity groups.** The neutrophil% in all comorbidity groups was analyzed by One way ANOVA and Kruskal-Wallis test (p=0.4001). Post-hoc analysis by Dunn’s multiple comparison test (all comparison is not significant). Group 0-No comorbidities, 1-Heart failure (LVEF<50%), 2-CAD, 3-Pulmonary HTN (static sPAP>35), 4-Lung cancer, 5-Anxiety/Depression, 6-Osteoporosis, 7-Malnutrition (BMI<20), 8-DM, 9-OSA, 10-Normocytic anemia, 11-Lung fibrosis. The full-length immunoblot images are included in additional file 3.
