# Supplementary material for: Suppressor of variegation 3–9 homologue 1 impairment and neutrophil-skewed systemic inflammation are associated with comorbidities in COPD
Source: BMC Pulm Med. 2021 Oct 2;21:276. doi: 10.1186/s12890-021-01628-x (PMC8487160; doi:10.1186/s12890-021-01628-x)
Supplement: Supplementary file 2 — Additional file 2: Table 1. Characteristics of the study subjects with normal or COPD for immunoblot assays. [file 12890_2021_1628_MOESM2_ESM.docx]

| **Supplementary Table 1. Characteristics of the study subjects with normal or COPD for immunoblot assays.** | | | | | | |
| --- | --- | --- | --- | --- | --- | --- |
| **Characteristic** | **Normal** | |  | **COPD (** **GOLD stage)** | | |
|  | **Non-smoking** | **Smoking** |  | **I or II** | | **III or IV** |
| **Subjects (n)** | 6 | 7 |  | 19 | | 11 |
| **Age years**  **(median, IQR)** | 70.00 ± 3.03  (70.00, 4.75) | 70.00 ± 3.41  (70.00, 4.00) |  | | 70.32 ± 8.68  (72.00, 13.50) | 68.18 ± 7.74  (67.00, 12.00) |
| **Sex M/F (%)** | 5(83.33) / 1(16.67) | 6(85.71) / 1(14.29) |  | 18(94.74) / 1(5.26) | | 11(100) / 0(0) |
| **BMI kg∙m^-2^**  **(median, IQR)** | 25.49 ± 4.70  (24.52, 5.53) | 26.93 ± 6.65  (23.81, 6.33) |  | 24.02 ± 3.33  (24.03, 3.69) | | 21.83 ± 3.03  (21.82, 3.99) |
| **Smoking Yes/No (%)** | 0(0) / 6(100) | 7(100) / 0(0) |  | 17(89.47) / 2(10.53) | | 9(81.82) / 2(18.18) |
| **FEV1/FVC%**  **(median, IQR)** | 102.50 ± 13.69  (100.00, 11.25) | 107.14 ± 16.33  (110.00, 20.00) |  | 61.47 ± 5.42 ^a,b^  (63.00, 4.75) | | 46.18 ± 14.70 ^a,b,c^  (42.00, 23.50) |
| **FEV1%**  **(median, IQR)** | 100.83 ± 2.48  (100.50, 3.75) | 106.71 ± 3.33  (106.00, 5.00) |  | 69.51 ± 14.51 ^a,b^  (66.00, 17.90) | | 30.75 ± 9.80 ^a,b,d^  (27.00, 11.80) |
| Data are expressed as n, mean±SD, or percentage, median and interquartile range (IQR) in bracket. COPD: chronic obstructive pulmonary disease; GOLD: Global Initiative for Chronic Obstructive Lung Disease; M: male; F: female; FEV1: forced expiratory volume in 1 s; FVC: forced vital capacity, BMI: Body mass index. The values of FEV1/FVC% and FEV1% were analyzed by Tukey's multiple comparison tests (^a,b^*p*<0.01, COPD patients compared with non-smoker or smoker; ^c^*p*<0.05, ^d^*p*<0.001,COPD patients with stage I/II compared with stage III/IV). | | | | | | |
